# *Leishmania* mediated regulation of host metabolism: impact on host immunity

**DOI:** 10.3389/fimmu.2026.1750304

**Published:** 2026-02-18

**Authors:** Somtochukwu S. Onwah, Gaurav Gupta, Romaniya Zayats, Amol Singh Banga, Kashish Chhotaria, Gurtej Ahluwalia, Jude E. Uzonna

**Affiliations:** 1 Department of Immunology, Max Rady College of Medicine, University of Manitoba, Winnipeg, MB, Canada; 2 Biotechnology and Bioinformatics, NIIT University, Neemrana, Rajasthan, India; 3 Department of Medical Microbiology & Infectious Diseases, Max Rady College of Medicine, University of Manitoba, Winnipeg, MB, Canada; 4 Department of Pathology, Max Rady College of Medicine, University of Manitoba, Winnipeg, MB, Canada

**Keywords:** host parasite, immune cells, immune response, Leishmania, metabolism

## Abstract

Leishmaniasis, a vector-borne disease affecting millions worldwide, is caused by protozoan parasite *Leishmania*. In mammalian hosts, *Leishmania* survives in the hostile environment of macrophages by exploiting key metabolic pathways to evade their destruction and subvert the host immune responses. Understanding of how *Leishmania* alters host immune cell metabolism could reveal novel targets for vaccines and therapeutics for effective control of Leishmaniasis. This review focuses on *Leishmania*-induced modulation of host immune response, with particular focus on reprograming of key metabolic pathways in macrophages, dendritic cells, T cells and other immune cells.

## 1 Introduction

Leishmaniasis is a vector-borne disease that threatens approximately 350 million individuals globally. It is caused by the protozoan parasite *Leishmania*. In mammalian hosts, the parasites survive and proliferate within macrophages. The disease manifests in three main clinical forms: visceral leishmaniasis (VL), cutaneous leishmaniasis (CL), and mucosal leishmaniasis (ML). These clinical forms are based on the infecting *Leishmania* species and strain, parasite virulence, and immunological status of the host (1).

Visceral leishmaniasis (VL), the most severe form of the disease, is caused by *Leishmania donovani* in the Old World and *Leishmania infantum* in the New World. It is associated with significant clinical symptoms, including hepatosplenomegaly, and can be fatal if left untreated (2). Cutaneous leishmaniasis (CL) is the most common form of the disease and is caused by several species, including *L. major*, *L tropica* (Middle East and Mediterranean Region), *L. mexicana* (Central America), *L. amazonensis* (South America), and *L. aethiopica* (old world). The form is characterized by localized skin lesions that heal slowly. Although lesions may resolve spontaneously, they can progress into chronic conditions, resulting to severe tissue damage and disfigurement. In immunocompromised patients, lesions may disseminate, resulting in diffuse cutaneous leishmaniasis (DCL) (3, 4). Mucocutaneous leishmaniasis (ML), caused primarily by *L. braziliensis*, is endemic in South America, and is characterized by chronic inflammation and extensive tissue destruction of the nasal and oropharyngeal mucosa (3).

Treatment of leishmaniasis involves the use of several pharmacological agents, including amphotericin B, pentavalent antimonial medications, paromomycin, and miltefosine. Treatment regimen ranges from localized therapies for cutaneous lesions to systemic, often toxic approaches for DCL, ML, and VL (5–8). The ability of *Leishmania* parasites to evade the host immune response and persist despite treatment has prompted a reevaluation of current therapeutic strategies (9). *Leishmania* exploits some host metabolic pathways such as arginine, thiol, and polyamine syntheses to thrive in the hostile environment of the host macrophages. Immune cells such as macrophages, neutrophils, T cells, etc play a critical role in the host immune response to *Leishmania.* Here in this review, we will discuss the regulation of immune cell functioning by *Leishmania* or *Leishmania* derived metabolites, that favors its survival and promotes disease pathogenicity.

## 2 Metabolic adaptations in *Leishmania*

*Leishmania* has evolved metabolic adaptions that enable survival in distinct dimorphic forms across its complex life cycle. The promastigote and amastigote stages inhabit different hosts and therefore rely on distinct metabolic programs to successfully survive and proliferate (10–12). In this section, we discuss key metabolic pathways in *Leishmania* that facilitate host exploitation and parasitism.

### 2.1 L-arginine metabolism

*Leishmania* is critically dependent on L-arginine for survival (13) and possess its own L-arginine metabolic pathway (14, 15). The parasite imports its own L-arginine via the transporters LdAAP3 and CAT-2B (16), with LdAAP3 being highly specialized for arginine uptake under acidic conditions (16, 17). *Leishmania* also expresses key enzymes involved in L-arginine metabolism, including arginase (ARG), and ornithine decarboxylase (ODC) (13, 18–20). Notably, parasite arginase activity can be enhanced by the host protein, insulin-like development factor-I (IGF-I), thereby promoting parasite growth in macrophages (21). ARG converts L-arginine to L-ornithine, which is subsequently metabolized by ODC to polyamines: putrescine, spermidine and spermine, that are essential for parasite proliferation (19).

Given its importance for parasite survival, the L-arginine metabolic pathway represents a key target of host defense (10). Pharmacological inhibition of arginase, such as using N^ω^-hydroxy-l-arginine, leads to a decrease in parasite growth (22). Similarly, treatment of *Leishmania-*infected macrophages with arginase inhibitor decreases intracellular parasite burden by suppressing both host and parasite arginase activity (23). Likewise, ODC inhibitors, such as D, L-*α*-difluoro-methyl ornithine (DFMO), and 3-aminooxy-1-aminopropane (APA), restrict parasitic infection (24–26). Together, these findings support the concept that *Leishmania* has evolved its L-arginine metabolic machinery to compete with the host for this essential nutrient in order to survive and proliferate (10).

### 2.2 Polyamine pathway

Polyamines (PA), including putrescine, spermidine (Spd), and spermine, are produced via the ARG pathway, which is induced in alternatively activated and metabolically suboptimal macrophages (27, 28). These PA support parasite replication and persistence and are essential for trypanothione sysnthesis, a thiol-based antioxidant that protects *Leishmania* from peroxynitrite-mediated cytotoxicity (29). In the PA biosynthetic pathway, ARG converts L-arginine to L-ornithine, which is then decarboxylated by ODC to produce putrescine. Putrescine subsequently converted to spermidine by spermidine synthase (SpdS), using an aminopropyl group donated by decarboxylated S-adenosylmethionine, generated by S-adenosylmethionine decarboxylase (AdoMetDC) (30). Spermine is produced by the addition of a second aminopropyl group by spermine synthase (31).

Several PA biosynthetic enzymes are essential for *Leishmania* survival and represent promising novel drug targets. AdoMetDC deficient parasites are unable to grow in PA-depleted conditions, a phenotype rescued by spermidine but not by putrescine or spermine. Consequently, inhibitors of AdoMetDC, such as MDL73811 and CGP 40,215A display potent antileishmanial activity (32, 33). SpdS is also essential for *L. donovani* amastigotes, as *spds*^-/-^ parasites require exogenous spermidine for growth and exhibit reduced infectivity *in vivo* (31). Notably, pentamidine-resistant parasites adapt by increasing SpdS affinity for putrescine while reducing drug binding, highlighting the role of PA metabolism in drug resistance (33, 34).

### 2.3 Thiol metabolism

*Leishmania* maintains a redox homeostasis through a unique thiol-based system distinct from its invertebrate and vertebrate hosts. The parasite lacks glutathione reductase (GR) (35, 36) and instead relies on N^1^, N^8^-bis(glutathionyl) spermidine (trypanothione) and its precursor, N^1^-glutathionyl spermidine, as the principle intracellular thiols. These molecules are recycled by trypanothione reductase (TR), an NADPH-dependent flavoprotein oxidoreductase (35, 37, 38). Although structurally related to GR, TR has distinct substrate specificity, enabling selective pharmacological targeting (37, 39). TR protects *Leishmania* from oxidative stress and its deficiency markedly attenuates parasite virulence (36, 40, 41). Trypanothione further protects *Leishmania* by scavenging nitric oxide (NO) and labile iron by forming a di-nitrosyl iron complex (36, 42, 43).

Additionally, tryparedoxin-dependent peroxidases (TPs), which are members of the 2-cysteine peroxiredoxin family, protect *Leishmania* from hydroperoxides by reducing them to water and alcohol (44–46). TPs have also been implicated in the metastasis of *Leishmania guyanensis* (47), enhanced infectivity, and reduced parasite responsiveness to antimonial drugs (46).

### 2.4 Sterol metabolism

Ergosterol is essential for maintaining cell membrane integrity of fungi and protozoa, like the role of cholesterol in mammalian cells. Moreover, *Leishmania* cannot synthesize cholesterol and instead acquires it from the host cell (48). Amphotericin B (Amp-B), a key antileishmanial drug, has high affinity for ergosterol and induces parasite death by forming pores in the plasma membrane leading to its lysis (49). Dynamic changes in sterol composition have been linked to promastigote metacyclogenesis and altered parasite virulence (50).

Sterol alkylation position C24, catalyzed by the enzyme S-adenosyl-L-methionine: C24-**Δ**-sterol-methyltransferase (SMT), represents the final step of sterol biosynthesis in trypanosomatids. Inhibition of SMT affects parasites survival through sterol depletion leading to morphological defects (51). However, loss of functional SMT has also been associated with Amp-B drug resistance as parasites deficient in SMT lack ergosterol, reducing Amp-B insertion into the parasite membrane (52). Upstream of SMT, C14α-sterol demethylase (14DM) catalyzes demethylation at the C14 position of sterol intermediates and is the primary target of azole drugs, including itraconazole (ITZ) (20, 49). Depletion of 14DM in *Leishmania* promastigotes results in pronounced morphological and cytokinetic defects, although parasites retain the capacity to proliferate at a reduced growth rate (53).

### 2.5 Glucose metabolism

Glucose is a major carbon and energy source for *Leishmania* (54). Although both promastigote and amastigote forms utilize glucose, catabolism of this molecule is significantly higher in the promastigotes (55). This reflects the sugar rich microenvironment of the sandfly and glucose-limited phagolysosomes of the macrophages where promastigotes and amastigotes respectively reside (56, 57). Accordingly, amastigotes exhibit reduced glucose uptake and rely predominantly on fatty acid oxidation for energy production (55, 58). In this context, glucose uptake primarily supports the biosynthesis of glycoconjugates, including **β**-mannan. Genetic ablation of glucose transporters disrupts glycoconjugates biosynthesis and compromises parasite survival (59). In *L. mexicana*, glucose transporter deficient promastigotes (Δ*lmgt*) display reduced growth rates (60). While Δ*lmgt* amastigotes show severely reduced viability inside macrophages and fail to proliferate as axenic amastigotes, depicting the essential role of glucose transporters in amastigote survival (60).

Under oxidative stress, *Leishmania* redirects glucose metabolism from glycolysis toward the pentose phosphate pathway to restore NADPH levels. Overexpression of glucose-6-phosphate dehydrogenase (G6PDH) and transaldolase enhances resistance to reactive oxygen species (ROS) and antileishmanial drugs, including sodium antimony gluconate (SAG) (61). Consistently zinc sulfate exhibits direct antileishmanial activity by inhibiting multiple enzymes involved in glucose metabolism, including fructo-phosphokinase, glucose phosphate isomerase, hexokinase, G6PDH, and ribose-5-phosphate isomerase (62). Deletion of Phosphomannomutase (PMM) which catalyzes mannose-6-phosphate to mannose-1-phosphate in *L. mexicana*, results in loss of virulence, indicating identifying PMM as a promising therapeutic drug target (63).

### 2.6 Iron and heme metabolism

*Leishmania* lacks *de novo* heme biosynthesis and iron/heme storage system. Given the critical role of heme and iron in parasite survival and virulence, *Leishmania* has evolved efficient scavenging system to obtain these nutrients from the host (64–66). Hemoglobin degradation and heme depletion serves as differentiation cues, driving promastigote maturation from a proliferative, non-infective form to a non-dividing, infective metacyclic stage (67, 68). Amastigotes survive in the parasitophorous vesicle of a macrophage by scavenging host-derived heme and iron (69). Inside the parasitophorous vacoule, iron is primarily present as Fe^3+^ complexed with transferrin and lactoferrin. *Leishmania* preferentially utilizes Fe^2+^ which is generated through NADPH-dependent reduction of Fe³^+^ by *Leishmania* ferric reductase 1 (LFR1), which is expressed on parasite membrane (70–72). Uptake of reduced iron is mediated by *Leishmania* iron transporter 1 (LIT1), an iron-responsive transporter essential for parasite replication and virulence (68, 69) Similarly, *Leishmania* heme response 1 (LHR1), present in the plasma membrane and endocytic compartment, promotes heme transport to the cytosol and its absence attenuates parasite virulence (73, 74).

Following cytosolic uptake, iron is transported into mitochondria by *Leishmania* mitoferrin 1 (LMIT1) (75), where enzymes including coproporphyrinogen oxidase, protoporphyrinogen oxidase, and ferrochelatase, contribute to heme biosynthesis from host-derived precursors (66, 76, 77). Iron is also required in glycosomes, where anti-oxidant enzymes such as superoxide dismutase A (SODA) and ascorbate peroxidase (AP) depend on iron cofactors to protect parasites from oxidative stress within the macrophage environment (78, 79).

## 3 Metabolic changes in immune cells during *Leishmania* infection

*Leishmania* infects various host cells that play a crucial role in determining the outcome of infection, including monocytes, macrophages, and dendritic cells (DCs). Following uptake of the parasites, phagocytes internalize promastigotes into phagosomes, which then fuse with lysosomes. Remarkably, *Leishmania* is among the few protozoan parasites capable of surviving and replicating within this hostile environment (12, 80). This is facilitated by their modulation and/or metabolic reprogramming of infected cells. Understanding how *Leishmania* survives and drives metabolic reprogramming in host cells is critical for understanding disease pathogenesis and could inform new approaches to vaccine, drug, or therapeutic development. In the following discussion, we provide an overview of the mechanisms by which *Leishmania* modulates the activation of innate and adaptive immune cells by directly or indirectly influencing their cellular metabolism.

### 3.1 Macrophages

Macrophages play a dual role in *Leishmania* infection: they serve as the definitive hosts for parasite proliferation and as effector cells involved in parasite clearance. For parasite clearance, macrophages must be classically activated (M1), resulting in the production of parasiticidal effector molecules. This classical activation requires stimulation by interferon-gamma (IFN-γ), produced primarily by activated CD4^+^ T cells. The binding of IFN-γ to its receptors on macrophages induces the expression of inducible nitric oxide synthase (iNOS), leading to the production of NO. This NO, together with its reactive derivatives, including peroxynitrates, contribute to parasite killing inside infected macrophages (12, 80). Conversely, alternatively activated macrophages (M2), which are favored by Th2 cytokines, including IL-4 and IL-13, promote parasite survival and replication. These cytokines, particularly IL-4, enhance polyamine synthesis by upregulating L-arginine metabolism (12). The macrophage polarization induced by *Leishmania* infection depends on parasite species and strain. In visceral leishmaniasis, *L. donovani* infection in humans and *L. infantum* infection in dogs are associated with M2 macrophage polarization, characterized by increased expression of CD163, IL-10, and CXCL14 (81–83). In contrast, cutaneous leishmaniasis exhibits mixed macrophage responses: *L. braziliensis* and *L. major* preferentially induce M1 macrophages, whereas *L. amazonensis* promotes M2 polarization (84–86). Thus, the activation state of macrophages –M1 or M2 – critically influences the outcome of *Leishmania* infection, determining whether the parasite is eliminated or allowed to proliferate.

**Figure 1:**
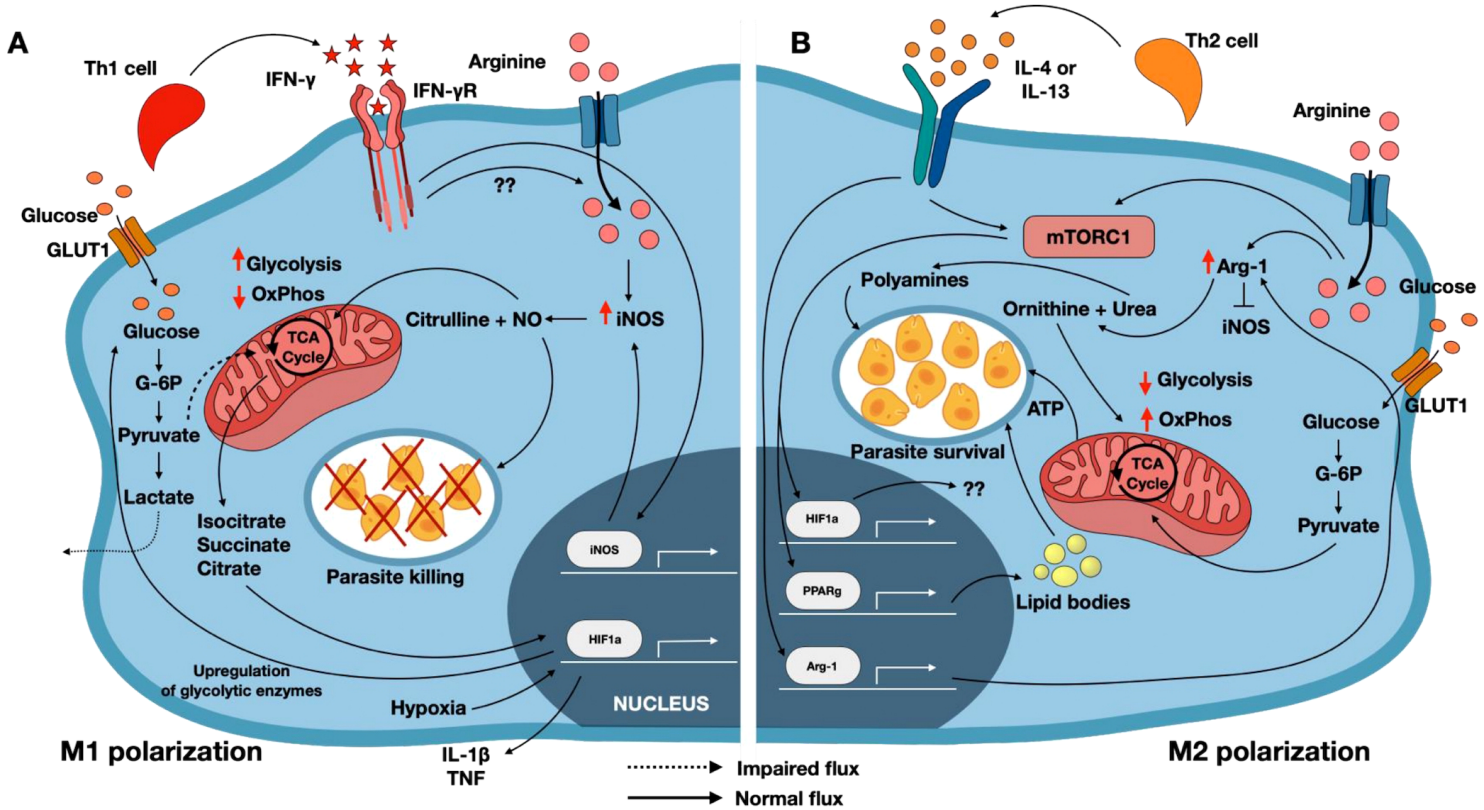
Metabolic reprogramming of macrophages metabolism during *Leishmania* infection. **(A)** Uptake of *Leishmania* promastigotes by macrophages triggers an influx of arginine into the cell. Concurrent ligation of IFN-γ and IFN-γR (driving M1 polarization) induces induction of iNOS leading to the production of nitric oxide (NO) and citrulline. NO diffuses into the phagolysosome to kill the parasites, and also enters the mitochondria, where it inhibits oxidative phosphorylation (OxPhos). Reduced OxPhos causes the accumulation of TCA cycle intermediates, including isocitrate, citrate and succinate, which stabilizes HIF-1α. Oxidative stress-induced hypoxia further enhances HIF1α activity, promoting glycolytic enzyme expression and increasing glycolytic flux. **(B)** In contrast, engagement of IL-4/IL-13 with their receptors (promoting M2 polarization) activates mTORC1 and induces Arg-1 expression which converts arginine into polyamines that promote parasite survival and support increased OxPhos. mTORC1 signaling promotes both HIF1α and PPARγ expression. In this context, the role of HIF1α remains unclear as it does not lead to enhanced glycolysis. PPARγ drives the formation of lipid bodies that promotes parasite survival. Arg-1 activity inhibits iNOS, reinforcing the M2 phenotype. iNOS, inducible nitric oxide synthase; mTORC1, mammalian target of rapamycin complex 1; Arg-1, Arginase; OxPhos, Oxidative phosphorylation; PPARγ, peroxisome proliferator activated receptor gamma; NO, nitric oxide; HIF1α, Hypoxia inducible factor 1alpha.

Apart from cytokines, the M1 or M2 activation state of macrophages is also influenced by metabolites in the cells’ microenvironment. Specifically, the levels of host amino acids and the status of central carbon metabolism play significant roles in determining macrophage activation state. Upon stimulation with IFN-γ, macrophages upregulate the cationic amino acid transporter, CAT2B, resulting in increased uptake of L-arginine and increased expression of iNOS (87, 88) (**Figure 1A**). iNOS in turn catalyzes the conversion of L-arginine to citrulline and NO, a potent parasiticidal molecule. The induction of iNOS and subsequent production of NO inhibits mitochondrial oxidative phosphorylation (OxPhos) (89), compelling macrophages to rely predominantly on glycolysis to meet their energy needs. This glycolytic shift supports the production of ROS, which supports M1 while inhibiting M2 macrophage polarization.

M2 macrophage polarization is driven by IL-4 and IL-13 signals through a heterodimeric receptor complex composed of the IL-4 receptor alpha chain (IL-4Rα) and the IL-13 receptor alpha 1 chain (IL-13Rα1) (90, 91) (**Figure 1B**). Ligation of the IL-4/IL-13 receptors leads to activation of the JAK/STAT6 signaling pathway, which induces the transcription of M2-associated genes, including CAT2B and Arginase-1 (Arg-1) (92). Arg-1 catalyzes the conversion of L-arginine to ornithine and urea, promoting polyamine synthesis that supports parasite proliferation. Arg-1 and iNOS compete for the same substrate, L-arginine, and their expression is reciprocally regulated by distinct cytokine cues. The upregulation of one enzyme can inhibit the function of the other; for example, Arg-1 inhibits iNOS, and vice versa.

Although host Arg-1 is a potent inhibitor of iNOS, it does not appear to contribute to play a significant role in disease pathogenesis (93). Genetic deletion of Arg-1 in both hematopoietic and non-hematopoietic in C57BL/6 mice had no noticeable effect on lesion development or parasite burden during *Leishmania* infection (93). Interestingly, pharmacologic inhibition of Arg-1 with nor-NOHA in BALB/c mice delayed disease progression, while ornithine treatment of the resistant C57BL/6 mice resulted in worsening of cutaneous leishmaniasis (94, 95). These findings suggest that both host-derived and parasite-derived Arg-1 activity may contribute to disease outcomes, possibly by increasing arginine metabolism and promoting parasite (95).

Following *Leishmania-*infection, murine macrophages initially develop a phenotype akin to an M1 state, characterized by elevated glycolysis levels and increased host glucose transporters (**Table 1**) (96). However, this proinflammatory phenotype is transient, because after 24 hours post-infection, they undergo a significant metabolic and functional shift toward an M2 phenotype, which is characterized by increased reliance on oxidative phosphorylation as studied by the Seahorse assay (96). This M2 polarization is supported by enhanced activity in the tricarboxylic acid (TCA) cycle, fatty acid β-oxidation, and glutaminolysis (**Table 1**). Additionally, M2 polarized macrophages exhibit increased expression of 3-phosphoglycerate dehydrogenase, which redirects glycolytic intermediates into serine, glycine and folate metabolism (108). The highly efficient OxPhos machinery in M2 macrophages results in elevated glucose levels, which in turn favors high intracellular glucose levels that promote the proliferation of intracellular amastigotes. In contrast, IFN-γ-activated M1 macrophages exhibit a metabolically rewired state that favors highly inflammatory glycolytic pathway. The internalized glucose is predominantly metabolized via the pentose phosphate pathway (PPP), generating NADPH and ATP, which are essential for the production of ROS. Although pyruvate is still produced, its entry into the TCA cycle is impaired, resulting in the accumulation of metabolites such as citrate, isocitrate and succinate in the cells. These intermediates promote β-oxidation of fatty acids and activate the transcription factor, HIF1α. HIF1α in turn promotes the induction of glycolytic enzymes and inflammatory cytokines, such as IL-1β, thereby reinforcing the microbicidal activity of M1 macrophages and contributing to parasite destruction (108).

**Table 1:** Steady and inflammatory metabolic states of immune cells and their corresponding effector function.

Immune cell	Steady state metabolism	Inflammatory state metabolism	Effector function	References
Macrophages		Glycolysis	M1- NO, ROS, which promotes parasite clearance of *L. infantum*.	(96)
		Oxphos, TCA cycle	M2- Arginase expression, which promotes parasite survival of *L. infantum*.	(96)
		Lipid body formation	M2- Lipid based inflammatory mediators: Prostaglandin PG2 Leucotriene LT4, which promotes parasite survival of *L. donovani*	(97)
Neutrophils	Glycolytic	Glycolysis	NADPH dependent NOX and NET induction, which might favor parasite clearance of *L. donovani*	(98–100).
		Mitochondrial oxPhos	Transmigration, which might favor parasite clearance	(98).
Dendritic cells	OxPhos and FAO	Aerobic glycolysis	Induction of HIF1α Reduction of IL-12 production TLR ligation, which favors parasite survival of *L. donovani* .	(101, 102).
Naïve T cells	OxPhos		DC interaction? Th cell differentiation	(103)
Th1 cells		Glycolysis Glutaminoysis	IFN γ production which favors parasite clearance	(104).
Th2 cells		Glycolysis OxPhos ROS	IL-4, IL-13, which usually favors parasite survival	(105)
T regulatory cells		OxPhos FAO	Foxp3 expression Immunosuppressive function, which favors parasite survival	(106)
B cells	Glycolysis Basal mitochondrial respiration	Glycolysis PPP	Antibody production, which might promote both parasite survival or clearance.	(107)

The mammalian target of rapamycin (mTOR) is a major regulator of carbon metabolism in eukaryotic cells. There are two functional types of mTOR complexes expressed in immune cells: mTOR complex 1 (mTORC1) and mTORC2, which differ in their affinities for rapamycin and are largely independent. mTORC1 has been linked to the regulation of *Leishmania*-infected macrophage polarization. Specifically, M2 polarization is promoted via mTORC1 activation following the ligation of IL4/IL13 receptors on macrophages. Engagement of these receptors activates the PI3K/protein kinase B (Akt) pathway, which in turn activates mTORC1. Activated mTORC1 in turn promotes transcription and translation of metabolic enzymes necessary to mediate the synthesis of metabolites favorable to parasite proliferation (109). In support of this, inhibition of mTORC1 in mice infected with *L. donovani* reduced parasite numbers in *ex vivo* macrophages and granuloma formation in the liver (110). Additionally, mTORC1 activation upregulates the expression of enzymes involved in fatty acid synthesis and lipid body formation, resulting in elevated lipid and triglyceride levels in splenic and hepatic macrophages from *L. donovani*-infected mice, as assessed by real time PCR and high performance thin layer chromatography (111). Notably, mTORC1 also promotes the expression of HIF1α, a key transcription factor that supports metabolic reprogramming in cells. *L. donovani* infection downregulates HIF1α expression and this was associated with an increased number of amastigotes in macrophages (111). Interestingly, HIF1α appears to have a dual role in regulating macrophage M1 and M2 polarization. In M1 macrophages, succinate accumulation stabilizes HIF1α, promoting proinflammatory gene expression, leading to parasite killing. The exact mechanism by which the same transcription factor mediates M2 and M1 polarization is not fully understood. Still, it has been suggested that mTORC1 activation occurs through proteolytic cleavage mediated by parasite-derived metalloprotease, gp63. The inhibition of mTORC1 in *L. major*-infected macrophages has been shown to induce type 1 interferon response, resulting in M1 polarization and impairment in the OxPhos system. This metabolic reprograming leads to the production and accumulation of TCA cycle intermediates such as citrate, itaconate and succinate, which subsequently activate and stabilize HIF1α and reinforce the inflammatory parasiticidal state (112, 113).

Bioinformatic and immunofluorescence studies have shown that lipid bodies are formed in the cytoplasm of murine and human macrophages during *Leishmania* infection and are commonly found around the parasitophorous vacuole (114, 115). They serve as an alternative carbon source for *Leishmania* amastigotes and as a stable source of polyunsaturated fatty acids. In addition to fueling parasite metabolism, lipid bodies also protect the parasites against oxidative stress (116), thereby promoting parasite survival. Importantly, lipid bodies contribute to the establishment of the M2 macrophage phenotype during *Leishmania* infection by serving as precursors for lipid mediators that regulate both inflammatory and anti-inflammatory activities through omega-3 and omega-6 pathways (**Table 1**). For instance, the level of prostaglandin E2 (PGE2), a potent anti-inflammatory lipid molecule that dampens Th1 responses and promotes M2 polarization, is increased in *L. donovani*-infected macrophages (97). PGE2 acts in part via activation of the peroxisome proliferator-activated receptor gamma (PPAR-γ), which a master regulator of lipid metabolism that acts upstream of mTORC1. PPAR-γ activation promotes the expression of genes associated with OxPhos and Arg-1 enzyme, both of which are characteristic of M2 macrophage phenotype (117). In line with this, activation of PPAR-γ in both cutaneous and visceral leishmaniasis has been associated with progressive disease, while its inhibition of genetic disruption leads to reduced parasite burdens in infected macrophages (117, 118). PPAR-γ expression can be induced by anti-inflammatory cytokines such as IL-4, IL-10, IL-13 as well as various lipid ligands, including polyunsaturated fatty acid (PUFA) and prostaglandin E. Indeed, *Leishmania*-derived PUFA has been shown to directly influence the polarization of macrophages toward the M2 phenotype (119). Collectively, these findings show how *Leishmania* employs multiple mechanisms to strategically manipulate various metabolic pathways, particularly through lipid body formation, PPAR-γ activation, and mTORC1 signaling, to favor M2 macrophage polarization and support parasite survival and replication.

### 3.2 Neutrophils

**Figure 2:**
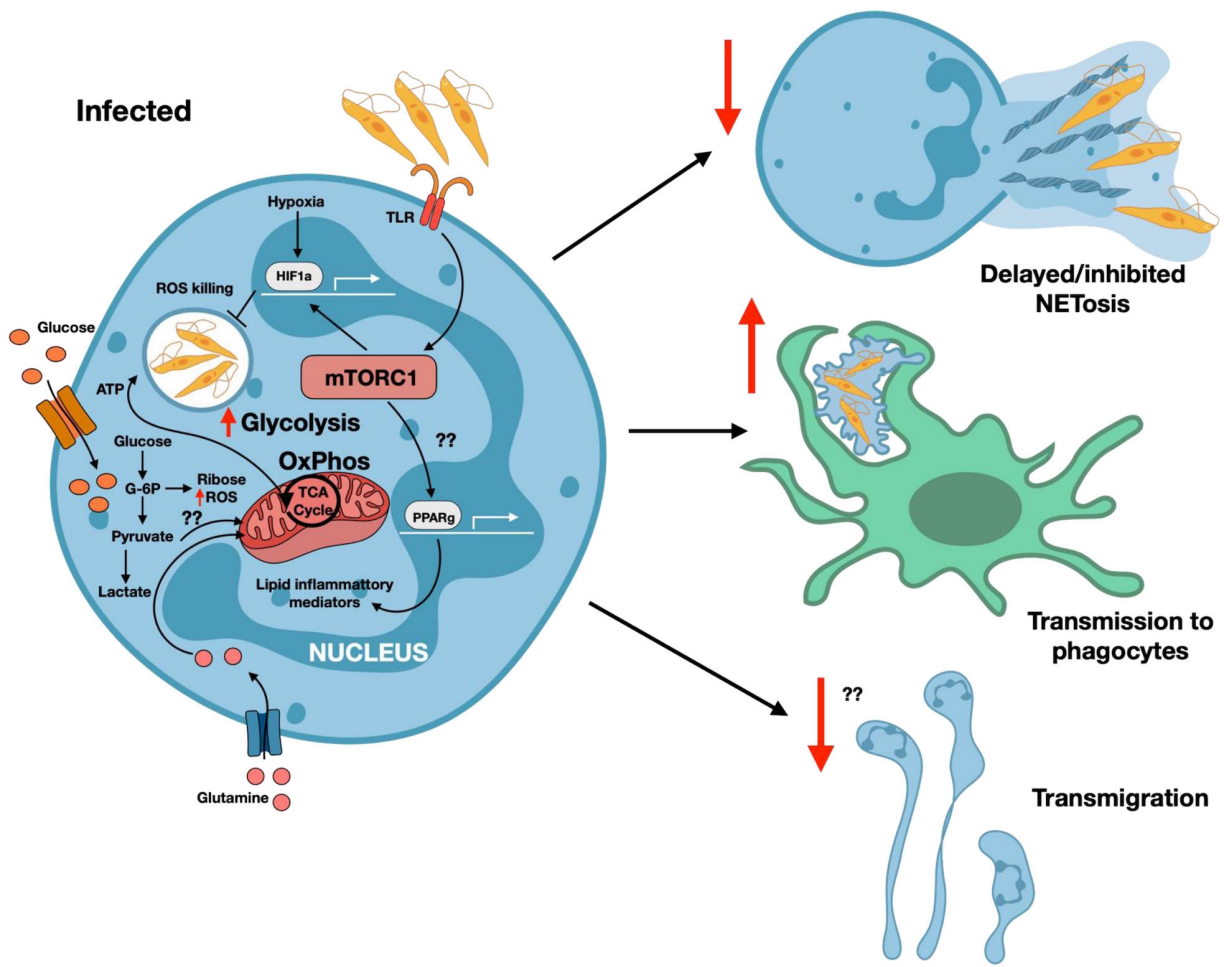
Metabolic reprogramming of neutrophils during *Leishmania* infection. Neutrophils normally rely on glycolysis for energy production under steady-state, oxygen-rich conditions supported by glucose and glutamine metabolism. Upon activation, particularly in the hypoxic microenvironment associated with *Leishmania* infection, neutrophils further increase glycolytic flux and enhanced activity of the pentose phosphate pathway (PPP) to generate NADPH required for reactive oxygen species (ROS) production and inflammatory mediator release. Activation promotes elevated glucose uptake and rapid conversion of glucose to glucose-6-phosphate (G6P), a critical metabolite that sustains glycolysis and supports neutrophil effector functions. As glycolytic activity intensifies, mitochondrial oxidative phosphorylation (OxPhos) becomes further reduced. Impaired OxPhos leads to the release and accumulation of tricarboxylic acid (TCA) cycle intermediates, which contribute to stabilization of HIF-1α. During *Leishmania* infection, both hypoxia and activation of the master metabolic regulator mTORC1 reinforce HIF-1α stabilization. mTORC1 also induces PPARγ activation, promoting the production of lipid-derived inflammatory mediators. Together, these metabolic shifts in neutrophil metabolism during *Leishmania* infection contribute to delayed or inhibited NETosis, which enables a ‘Trojan horse’ mechanism of parasites transmission to phagocytes. This occurs because *Leishmania* parasites resist neutrophil-mediated killings and impair neutrophil transmigration, enabling their survival and spread. ROS, reactive oxygen species; mTORC1, mammalian target of rapamycin complex 1; OxPhos, Oxidative phosphorylation; PPARγ, peroxisome proliferator activated receptor gamma; TCA, Tricarboxylic acid cycle; HIF1α, Hypoxia inducible factor 1alpha; TLR, Toll-like receptor.

Neutrophils are among the first immune cells recruited to sites of *Leishmania* infection and play a complex role in disease pathogenesis (120–122). *Leishmania* has evolved strategies to evade destruction by neutrophils including altering their metabolism to enhance its own survival. During their development and activation, neutrophils adapt to diverse and often harsh environments that are characterized by hypoxia and nutrient deficiency. This involves a metabolic reprogramming, transitioning from the bone marrow to a more activated states under disease conditions (98). Inflammation leads to recruitment of metabolically active immune cells resulting in localized hypoxia and reduced nutrient availability (123). Despite this, neutrophils retain functionality through metabolic adaptations that rely on glycolysis to supply over 90% of their ATP, independent of oxygen availability. This adaptation is augmented by activation of the transcription factor hypoxia-inducible factor (HIF-1α) which supports rapid ATP generation under hypoxic conditions (**Figure 2**). Under steady-state conditions, neutrophils primarily utilize glycolysis to generate ATP, and this is supported by high levels of oxygen, glucose, and glutamine present in plasma, bone marrow, and peripheral tissues (98). They also maintain a balanced redox state through the pentose phosphate pathway to produce NADPH, which is crucial for generating ROS for antimicrobial functions. This basal metabolic profile allows neutrophils to remain poised for activation without undergoing excessive and premature effector responses (124). Upon encountering inflammatory stimuli, neutrophils rapidly shift their metabolic profile to meet their increased energetic and biosynthetic demands. This includes increased glycolysis, enhanced PPP activity, and possibly increased fatty acid oxidation (FAO) (125). Collectively, these metabolic adaptations allow neutrophils to function efficiently in the diverse tissue environments.

Upon encountering *L. donovani* parasites, neutrophils undergo a metabolic shift characterized by increased glycolytic activity as measured by Seahorse analysis. This promotes the use of glucose to generate energy needed for its rapid response including phagocytosis, production of ROS, and release of inflammatory mediators (99). This allows neutrophils to combat the parasites at the site of infection, highlighting the crucial role of glucose metabolism in neutrophil-mediated immunity. Interestingly, inhibiting glycolysis negatively impacts the metabolic machinery of both *Leishmania* promastigotes and neutrophils, underscoring the dependency of both cells on glycolysis. Notably, no increase in ATP production from OxPhos was observed in *L. donovani*-infected neutrophils, further validating glycolysis as the dominant energy source in this context (99).

Under steady state condition, the uptake of glucose by neutrophils occurs primarily through the glucose transporter GLUT1 (126). Following activation, additional transporters, GLUT3 and GLUT4 are upregulated, ensuring sufficient glucose supply to meet the increased metabolic demands (127). Once internalized, glucose is rapidly phosphorylated to glucose-6-phosphate (G6P), preventing it from exiting the cell. G6P is then transported into the endoplasmic reticulum via the G6P transporter, where it can be converted back into glucose (98). This process is critical for restricting glucose metabolism fluxes in physiologic conditions, preserving energy metabolism for active inflammation.

The metabolic outcome of G6P (glycolysis or pentophosphate pathway) in neutrophils is influenced by their activation state. Glycolysis leads to the production of pyruvic acid, which, under aerobic conditions, can be further oxidized in the mitochondria via the TCA cycle (**Table 1**). However, liquid chromatography–mass spectrometry (LC–MS) and metabolic flux analyses reveal that, in activated neutrophils, pyruvate is predominantly diverted to lactate production, which is critical for sustaining glycolytic flux. Additionally, G6P in neutrophils also fuels the pentose phosphate pathway (PPP) metabolic process that generates NADPH which is essential for ROS-production (98, 128). Under homeostatic states, glutamine metabolism supports the basal synthesis of purines and nucleotides and contributes to the production of glutamate, aspartate, and lactate. However, during pathophysiological conditions characterized by reduced glucose levels, neutrophils increasingly rely on glutamine metabolism, which shift enhances NADPH production and NOX function (129). Despite reliance on glycolysis for energy, neutrophils still retain functional mitochondrial. The extent to which neutrophils retain mitochondrial metabolism remains understudied. Current evidence suggests that glucose-derived acetyl CoA plays a minimal role in mitochondrial ATP generation, while fatty acid-derived CoA may serve as an alternative substrate (130).

In neutrophils, mTOR functions as a central regulator of metabolic activity during inflammatory conditions. Loss of mTOR in activated neutrophils limits their ability to migrate, differentiate, and eliminate pathogens through the formation of neutrophil extracellular traps (NETs) (131, 132). Evidence suggests that *Leishmania* may modulate mTOR in neutrophils, thereby delaying or suppressing the execution of neutrophil effector functions. Additionally, hypoxia-inducible factor 1-alpha (HIF1α) is critical for neutrophil antimicrobial activity and survival; and its absence significantly reduces both functions (133). Another important metabolic regulator, peroxisome proliferator-activated receptor gamma (PPARγ), appears to influence effector function of neutrophils. In models of sepsis, elevated PPARγ levels appear to influence neutrophil chemotactic activity, and this effect could be reversed with the administration of a PPAR antagonist (134). Collectively, these findings suggest that *Leishmania* may impair neutrophil activation and oxidative effector functions by modulating key pathways of glucose metabolism. Further investigation is needed to fully understand how different *Leishmania* species modulate the activity of key metabolic enzymes in neutrophils.

### 3.3 Dendritic cells

DCs serve as a critical bridge between the innate and adaptive immunity. In leishmaniasis, DC indirectly influence macrophage polarization and parasite killing by enhancing Th1 polarization leading to IFN-γ production necessary for parasite killing (12). However, it is believed that *Leishmania* modulates DCs to subvert their functions and hence evade immune clearance.

**Figure 3:**
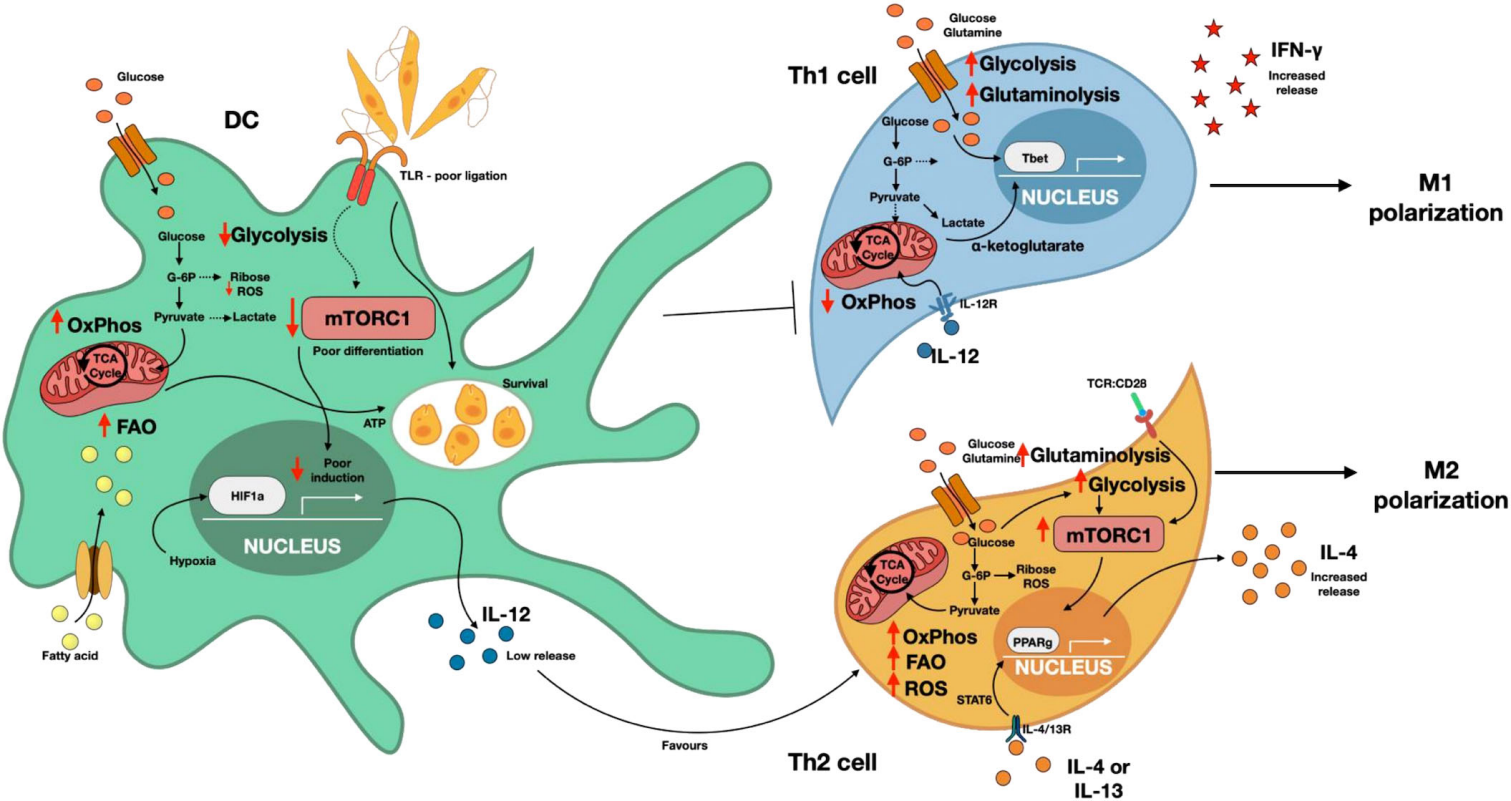
*Leishmania* reprograms DC metabolism to promote survival via induction of Th2 response.During *Leishmania* infection, impaired TLR signaling in dendritic cells (DCs) results in weak induction of mTORC1 and poor stabilization of HIF1α. Consequently, glycolytic flux is reduced, while oxidative phosphorylation (OxPhos) and fatty acid oxidation (FAO) were increased, generating ATP through the TCA cycle in a manner that supports parasite survival. Although inflammatory environments typically promote hypoxia and HIF1α stabilization, *Leishmania*-infected DCs exhibit inadequate HIF-1α induction, leading to low release of IL-12. Low IL-12 production favors the development of a Th2 response, promoting M2 polarization and simultaneously suppressing the Th1 response critical for M1 polarization. Th2 cells display elevated glycolytic flux, OxPhos, FAO and ROS production, all regulated through mTORC1-dependent induction of PPARγ. Through STAT6 signaling, PPARγ further enhances IL-4 production, reinforcing M2 polarization. In contrast, Th1 cells rely on increased glycolytic flux with reduced OxPhos, a metabolic profile driven by IL-12R ligation. TCA cycle metabolites such as α-Ketoglutarate, along with glycolytic intermediates, promote T-bet induction to increase IFN-γ production, ultimately favoring M1 polarization. ROS, reactive oxygen species; mTORC1, mammalian target of rapamycin complex 1; OxPhos, Oxidative phosphorylation; PPARγ, peroxisome proliferator activated receptor gamma; TCA, Tricarboxylic acid cycle; HIF1α, Hypoxia inducible factor 1alpha; TLR, Toll-like receptor.

DC immunometabolism is central to their effector activity. Under homeostatic conditions, DCs rely on oxidative phosphorylation (OxPhos) and fatty acid oxidation (FAO), whereas inflammation drives a shift in their metabolism toward aerobic glycolysis, which is vital for initiating inflammatory responses (135). Upon TLR ligations DCs significantly increase their glucose uptake and lactate production (101). Early after activation, pyruvate from glycolysis powers the TCA cycle and OxPhos to generate ATP (136). However, around 12 hours post-activation, NO production suppresses OxPhos, forcing reliance on glycolysis for ATP (137). Because glycolysis yields limited ATP, activated DCs engage in complementary pathways as fatty acid synthesis (FAS) and the pentose phosphate pathway (PPP) to support biosynthetic demands (101). Transcriptional profiling indicates that energy production in DCs containing glutaraldehyde-fixed *Leishmania* depends on TCA cycle and OxPhos. The parasite might also modulate host lipid metabolism by increasing cholesterol uptake while suppressing efflux, leading to cholesterol accumulation within infected DC (**Figure 3**) (138).

The inflammatory environment is usually hypoxic, which triggers the upregulation and stabilization of HIF1α promoting transcription of glycolytic enzymes (139). Interestingly, mTOR dependent HIF1α activation is independent of glucose availability and sustains DC activation following TLR ligation (101, 140). In visceral leishmaniasis, elevated HIF1α expression in splenic DCs correlates with reduced IL-12 production and impaired Th1 cell expansion (102). Although HIF1α promotes increased glycolytic flux (**Table 1**), it does not necessarily optimize DC effector function. mTORC1 also supports DC differentiation, and its inhibition leads to a reduction in DCs numbers and IL-12 production, thereby weakening the Th1 immune response (141, 142).

Overall, the suboptimal effector activity of DCs during *Leishmania* infection may reflect parasite-driven modulation of mTORC signaling. Although the role of DC immunometabolism in *Leishmania* infection is just starting to be defined, elucidating these pathways will be key to fully understanding and restoring DC-mediated immunity during infection.

### 3.4 T-cells

During *Leishmania* infection, in addition to classical Th1 and Th2 cells, other CD4^+^ T helper cell subtypes, such as Th9, Th17, T-regulatory cells (Tregs), and T follicular helper cells (Tfh), can also be induced (143). Th1 cells secrete IFN-γ, which activates infected macrophages to produce ROS and NO, promoting intracellular parasite clearance. Conversely, Th2 cells produce IL-4, IL-5 and IL-13 which drive alternative macrophage activation, increase arginase activity, and promote parasite survival (80). Here, we focus on metabolic reprogramming in T helper cells and Tregs, emphasis on how their interactions with DCs influence their effector functions.

Quiescent naïve T cells, recently matured in the thymus following clonal selection, primarily rely on OxPhos to meet energy demands, occasionally supplementing with glycolysis during intermediate stages after T cell receptor (TCR) rearrangements (**Table 1**) (103). Upon encountering antigen via TCR-MHC engagement and costimulatory molecules in peripheral tissues, naïve T cells undergo significant metabolic reprogramming, shifting toward aerobic glycolysis while maintaining sine pyruvate flux into the TCA cycle to generated biosynthetic intermediates for lipid or nucleotide synthesis (144). This anabolic shift supports the high metabolic demands of cell growth, proliferation and effector function. For example, glycolytic intermediates such as G6P and 3-phosphoglycerate can be diverted to the PPP and serine synthesis pathway, facilitating nucleotides and amino acids synthesis that is critical for continuous cell activation (145). Elevated glycolytic activity in activated CD4^+^T cells drives the proliferation and differentiation of multiple effector T cell subtypes, including Th1, Th2, Tfh, Treg and Th17 cells.

#### 3.4.1 Th1 cells

Th1 cells rely on both glycolysis and glutaminolysis for proliferation. α-ketoglutarate, a byproduct of glutaminolysis, promotes Tbet expression and enhances mTORC1 signaling, thereby promoting Th1 differentiation as assessed using Seahorse XF24 Extracellular Flux Analyzer (146). Glutamine availability is essential for lineage commitment of naïve CD4^+^ T cells. In its absence, naïve CD4+ T cells preferentially differentiate into FoxP3^+^ Tregs even under Th1 polarizing conditions (146). Th1 proliferation and differentiation also depend on amino acid availability, with L-amino acid transporter (LAT1) being essential for both Th1 and Th17 differentiation *in vitro*. In human Th1 cells, the complement receptor, CD46, regulates amino acid and glucose uptake, linking the complement system to metabolic programming (147).

Aerobic glycolysis is directly linked to IFN-γ production by Th1 cells (**Table 1**). Chang et al, showed that glyceraladehyde 3-phosphate dehydrogenase (GAPDH), binds to the 3’ UTR of *ifn-*γ mRNA to repress IFN-γ translation. However, during glycolysis, this binding is reversed, enabling normal IFN-γ production (148). Lactate dehydrogenase A (LDHA), highly expressed in Th1 cells, sustains aerobic glycolysis but does not bind to the 3’ *ifn-*γ UTR. Instead, LDHA influences IFN-γ-expression epigenetically. LDHA-deficient Th1 cells exhibited reduced histone H3Hk9 acetylation at the *ifn*-γ locus. LDHA deficiency diverts pyruvate to the TCA-cycle, reduces histone H3K9 acetylation and impairs Th1 immunity (148). In the context of *Leishmania* infection, glucose scarcity may limit glycosis in Th1 cells, resulting in reduced IFN-γ production and enhanced parasite survival. Moreover, intramacrophage lipid accumulation and a high fat diet result in defective *Leishmania*-specific Th1 cell response, increasing susceptibility in *L. donovani* infected mice (149). However, further studies are required to fully understand how *Leishmania* manipulates host lipid metabolic enzymes to subvert Th1 immunity.

#### 3.4.2 Th2 cells

Susceptibility in *Leishmania* infection is linked to Th2 cell expansion. Polarized Th2 cells produce IL-4, IL-13 and IL-5, which drive M2 macrophage activation. This process also promotes the upregulation of arginases, enhancing parasite proliferation and survival through the production of polyamines (80). Similar to Th1 cells, Th2 cells metabolically rely on glycolysis for their function. However, they also exhibit features of oxidative metabolism. Th2 cytokines activate STAT6-dependent activation of PPAR receptors on macrophages, promoting oxidative metabolism (150). In Th1 and Th2 cells, PPAR activation enhances the uptake of fatty acids via the TCR/CD28-mTORC1 axis as assessed by FACS analysis of Bodipy dyes used for identifying lipids and fatty acids (151). Indeed, PPAR deficiency has been shown to abolish IL-4 and IL-13 (Th2) responses in allergen and helminth infection models (105). Although the relative contributions of mTORC1 or mTORC2 in regulating glycolytic metabolism of Th2 cells remains debated, deletion of mTORC2-regulated GTPase RhoA reduces glycolytic flux and decreased production of IL-4 in Th2 cell in a mouse model of allergic asthma as assessed by Seahorse analysis and flow cytometry (152).

Overall, Th2 cells appear to utilize a mixed metabolic program, integrating fatty acid oxidation (FAO) and glycolysis to support differentiation, proliferation, and maintenance of effector functions in peripheral tissues (**Table 1**). Interestingly, ROS generated during mitochondrial oxidative phosphorylation are abundant under inflammatory conditions and can bias immune responses toward Th2 polarization by suppressing Th1 differentiation (153). In *Leishmania* infection, oxidative stress in genetically or immunologically susceptible hosts may thus promote Th2 dominance. However, the mechanisms by which *Leishmania* modulates Th2 glycolytic and FAO pathways to enhance survival remain poorly defined.

#### 3.4.3 Tregs

A notable feature of cutaneous leishmaniasis (CL) is that, even if resistant animals resolve the disease, sterile immunity is never achieved. Parasite persistence is maintained in part by Tregs, which act at the site of infection in a CCR5-dependent manner to suppress the effector CD4^+^ T cell responses (154, 155). Depletion of Tregs results in sterile immunity, but simultaneously abolishes the host’s natural acquired immunity to reinfection (154).

Tregs have a unique metabolic profile, relying predominantly on OxPhos from FAO rather than ATP generated through glycolysis (**Table 1**) (156). Foxp3, which is the master regulator of Treg cell development and function, enhances OxPhos activity and electron transport chain protein expression as shown by a Seahorse XF24 and XF96 analyzer, thereby enhancing the immunosuppressive activities of Treg cells (157). Mechanistically, Foxp3 binds to the TATA box of the *myc* gene and inhibits its glycolytic regulatory function (157). Elevated glycolytic activity in FoxP3^+^ Tregs results in a dysregulation of their suppressive function (106). Interestingly, GLUT1 expression is downregulated in murine iTregs, limiting glucose uptake and thereby favoring Treg polarization (156). Ironically, while glycolysis promotes proliferation of iTregs, it abrogates their immunosuppressive functions (158), highlighting distinct metabolic requirements versus function. Similarly, amino acid metabolism influences Treg differentiation because glutamine deprivation promotes Treg polarization even under strong Th1 polarizing conditions (146), while loss of amino acid transporters Sic7a5 or Sic1a5 impairs Th1, Th2 and Th17 differentiation without affecting Tregs (104). Tregs can also modulate DCs amino acid metabolism by limiting availability of essential amino acids, and this in turn enhances their survival and immunosuppressive function (159). The reliance of Tregs on FAO not only supports OxPhos-driven NAD^+^ production for TCA cycle activity but also protects them against fatty acid-induced cell death (157, 160). In *Leishmania* infection, inflammatory environments with limited glucose availability may promote Foxp3 expression in T cells leading to Treg expansion. This may contribute to the dampening of the adaptive immune response and maintenance of persistent parasites at the site of infection.

### 3.5 B-cells

In *Leishmania* infection, a hallmark B cell response is the induction of hypergammaglobulinemia. This is driven by B cells polyclonal activation, resulting in the production of large amounts of low-affinity anti-*Leishmania* antibodies and the formation of multiple immune complexes (161). Depending on the experimental model and/or parasite strain, B cells can play either protective or pathogenic roles in *Leishmania* infection (162–164).

Prior to antigen encounter and CD40-B-cell activating factor (BAFF) interactions, naïve B cells exhibit high glycolytic capacity and increased basal mitochondrial respiration (165). Antigen encounter through ligation of B cell receptor (BCRs) and/or CD40-IL-4 signaling triggers robust proliferation, protein synthesis and metabolic reprogramming (166). Activated B cells show enhanced glucose uptake via increased GLUT1 expression, leading to increased glycolytic activity, and partial diversion of the flux to the PPP pathway to generate NADPH (**Table 1**) (107). Concurrently there is upregulation of glycolytic enzymes and amino acid-metabolizing enzymes such as ornithine and phosphoserine aminotransferases, with peak expression observed at 3 days post-activation by Real Time PCR analysis. Increased amino acid uptake supports elevated alanine and glutamate production (167), while glycolytic flux into the hexosamine pathway facilitates antibody glycosylation in plasma cells as assessed by Seahorse analysis (168). Antibody synthesis in plasma cells imposes a high folding and unfolding load in the ER, generating stress responses and ROS. However, this oxidative stress responses are mitigated by increased glycolytic activity and PPP shunt (169). Immunometabolic regulation of activated B cell occurs through HIF1α, Gsk3, Myc and the TRAF3-NF-kB pathways (170). Although the precise mechanism of *Leishmania*-induced polyclonal activation of B cells remains unclear, parasite proteins LmSIR2 and LmS3a have been implicated in driving B cell glycolysis under inflammatory conditions (171).

## 4 Modulatory effects of parasite molecules on immune cells

*Leishmania* has a diverse repertoire of virulence factors, including A2 protein, glycoprotein 63 (GP63), lipophosphoglycan (LPG), release of extracellular vesicles (EVs), etc, that collectively modulate host immune cells to facilitate survival and persistence.

EVs released by *Leishmania* play a key role in infection and immunomodulation. Macrophages exposed to *L. amazonensis* EVs containing GP63 and LPG, show increased IL-6 and IL-10 expression. *In vivo* studies further demonstrate that *Leishmania*-derived EVs exacerbate the disease by skewing immune responses toward a Th2 phenotype (172). Additionally*, L. amazonensis* EVs induce NET formation, which may amplify inflammatory pathology (173). These EVs carry parasite specific proteins, lipids, and nucleic acids that can reprogram host cell metabolism (174, 175). EV cargo directly alters macrophage function by enhancing arginase-1–mediated urea and polyamine production, thereby skewing macrophages toward an M2 phenotype (175).

A2 protein, a crucial stress-induced virulence factor expressed in the endoplasmic reticulum, is essential for intracellular parasite survival in macrophages particularly at higher temperatures associated with visceral infection. A2 is expressed in both promastigote and amastigote stages of *L. donovani* but not in *L. major* (176, 177). Infection of neutrophils with A2-expressing *L. donovani* promastigotes promote neutrophil enlargement, apoptosis and transdifferentiation into DC-like phenotypes, facilitating parasite transmission and dissemination (178). Differential A2 expression in amastigotes also contributes to tissue tropism of parasites seen in post-kala-azar dermal leishmaniasis (PKDL) (176, 177, 179). Immunization studies demonstrate that A2 proteins induces robust T cells responses, including CD4^+^ and CD8^+^ T cell proliferation and IFN-γ (180, 181). Given the central role of A2 protein in stress response, amastigote survival, and visceralization (176, 177, 179), this protein likely contributes to hijacking host cell metabolic machinery and warrants further investigation.

GP63 is a highly expressed zinc metalloprotease that facilitate parasite entry and survival within macrophages by disrupting host signaling pathways required microbial activity (182). GP63 inhibits oxidative burst and chemotaxis of neutrophils, thereby early innate responses (183). In DCs, GP63 suppresses IL-12 production by activating host phosphatases (184). Additionally, GP63 also downregulates MHC class II, CD40 and CD86 expression, thereby limiting effective T cell activation (185). GP63 also cleaves CD4 molecules on T cells, impairing recognition of MHC class II restricted antigens (186). Beyond immune evasion, GP63 also targets metabolic and signaling pathways, including mTOR,4E-BP1, NF-κB, JAK/MAPK, and disrupts iron homeostasis via DICER1/hepcidin axis to favor parasite survival and replication (182, 187–189).

LPG is another crucial *Leishmania* virulence that interferes with host cell function. In macrophages, it inhibits phagosome maturation by preventing phago-lysosomal fusion (190), suppresses oxidative burst (191), NO synthesis (192) and IL-12 secretion (193) thereby creating a permissive intracellular niche for the parasite. LPG also impairs DC migration and IL-12 production (194). As a glycolipid antigen, LPG can stimulates T cell proliferation and IFN-γ production, highlighting its dual immunomodulatory role (195, 196). More recently, LPG has been shown to reprogram macrophage metabolism by promoting mitochondrial proliferation and enhancing oxidative phosphorylation, thereby supporting parasite survival (197).

Collectively, these parasite-derived molecules drive immune evasion, and modulate host cell response and metabolic reprogramming, thereby shaping host responses and disease outcome.

## 5 Effect of parasite metabolism on host cellular responses during *Leishmania* infection

The parasite’s metabolome greatly influences host cellular metabolic responses during *Leishmania* infection. Many studies examining the impact of parasite metabolism on host cellular immune response have utilized gene-deficient parasites. Polyamines, which are essential for parasite proliferation within macrophages, are synthesized by both host and parasite arginases, which limit NO production, thus favoring more polyamine synthesis. Parasite-specific arginases are particularly important for infectivity and disease pathogenesis (198, 199). Arginase-deficient *L. major* parasites display poor survival in macrophages, yet elicit immune responses (Th1 and Th2 cytokines) in Balb/c mice (198). While studies with arginase-deficient *L. mexicana* parasites showed poor survival in macrophages via increased iNOS2 activity (199). These findings suggest that parasite specific arginase, as in case of *L. mexicana* may influence macrophage metabolism, from a glycolytic-dependent M1 polarization to an OxPhos M2-like phenotype, rather than directly contributing to differentiation of Th1 and Th2 cells. In resistant mice, parasite-specific arginase appears to play a key role in regulating the induction of programmed cell death-1 (PD-1) expression on CD4^+^ T cells (200). Infection with arginase-deficient parasite in C57BL/6 mice increased PD-1-directed clonal exhaustion leading to impaired IFN-γ production (200), suggesting that parasite-specific arginase mitigates T cell exhaustion, thereby enabling the maintenance of M2 macrophage polarization.

Recently, our group has demonstrated that two key gluconeogenic enzymes of *Leishmania*, dihydrolipoyl dehydrogenase (DLD) and phosphoenolpyruvate carboxykinase (PEPCK), play a role in modulating host immunity during infection (201, 202). We found that CD4^+^ T cells specific for either DLD and PEPCK underwent robust clonal expansion, produced polyfunctional cytokines such as IFN-γ and TNF-α, and mounted strong memory responses upon secondary *Leishmania* challenge (201, 202). Although the expression of both enzymes occurs across all parasite life stages (202), gluconeogenesis predominates during the amastigote life stage, whereas promastigotes rely primarily on glycolysis (203, 204) The absence of PEPCK (by targeted gene deletion) in *L. major* impaired both promastigote and amastigote proliferation and subsequent cutaneous lesion development *in vivo* (205). Because *Leishmania* replicates within macrophages in the host, we hypothesize that reduced replication of PEPCK-deficient parasites in mice reflects, at least in part, PEPCK’s influence on macrophage activation and innate function. Although polarization of PEPCK-deficient *L. major*-infected macrophages was not assessed, the data suggests that the absence of PEPCK in *L. major* may shift macrophage metabolism from an M2 to an M1 phenotype. This assumption is supported by our finding of an increased Th1/Th2 cytokine ratio in mice infected with PEPCK-deficient parasites (205). Similarly, we recently demonstrated that in the absence of DLD- a critical component of the pyruvate dehydrogenase complex responsible for driving energy metabolism (206), parasite proliferation inside macrophages was severely impaired, resulting in the blunting of the host immune responses (11). Nevertheless, vaccination with these deficient parasites appeared to protect against virulent *Leishmania* infection by inducing a robust Th1-mediated host immune response (11). Interestingly, PEPCK-deficient parasites exhibited reduced ATP production and impaired OxiPhos (205). In addition, mitochondrial ultrastructure and function, including ROS production, membrane permeability, and oxygen consumption, were significantly impaired in DLD-deficient parasites (11). Such metabolic changes that alter ROS production (136) can, in turn, promote M1 macrophage polarization state. Further studies are needed to define the precise metabolic and immunological pathways through which PEPCK or DLD regulates macrophage phenotype during *Leishmania* infection.

### 5.1 Host-parasite nutrient competition

The phagolysosome, the intracellular niche for *Leishmania*, is a nutrient-limited environment in which host and the parasite compete for essential nutrients. During infection, macrophages restrict nutrient availability, forcing the parasite to scavenge for essential nutrients by expressing specialized sensors and transporters (207). This process, broadly termed nutritional immunity, is best illustrated by arginine metabolism. Because arginine is a substrate for both the host iNOS and arginase, competition for this amino acid represents a tug-of-war. Increased arginine flux through iNOS, depletes. Intracellular arginine, thereby limiting parasite proliferation (208). In response, the parasites sense arginine scarcity through mitogen-activated protein kinase 2 (MAPK)-mediated arginine deprivation response, enabling uptake of residual arginine within the phagolysosome (209, 210).

Aside from arginine, *Leishmania* is auxotrophic for several amino acids and carbon sources. Under nutrient limiting conditions, parasites enter a metabolically quiescent “stringent response”, characterized by reduced growth rate and metabolic reprogramming to ensure maximal utilization of scarce nutrients (211) through expression of parasite-specific transporters or sensors (212).

Iron is a critical micronutrient for antioxidant defense, electron transport and DNA synthesis. *Leishmania* lacks *de novo* iron synthesis and storage capacity and thus relies on scavenging iron from the host (64). Within the parasitophorous vacuole (PV), iron availability is tightly regulated by natural resistance-associated macrophage protein-1 (NRAMP-1), which restrict microbial access to iron by exporting it from the compartment (213). To compete for scarce iron, *Leishmania* employs specialized uptake systems, including ferric iron reductase (LFR1), ferrous iron transporter (LIT1) and the heme transporter1 (LHR1). LFR1 reduces Fe^3+^ to Fe^2+^, enabling LIT1-mediated cytosolic iron import, LHR1 facilitates heme acquisition from host hemoglobin to replenish parasite heme pools (64, 214). Although host iron homeostasis is normally regulated by ferritin and ferroportin, *Leishmania* can manipulate these pathways to increase iron availability. For instance, *L. amazonensis* infection promotes hepdidin-mediated degradation of ferroportin, thus limiting iron export from macrophage and increasing intracellular iron accessible to the parasite to siphon (214). This Ferroportin-hepcidin degradation pathway has been shown to increase parasite burden during infection (215). In addition to iron, *Leishmania* can exploit other host metal ions, including such Zn^2+^, Mg^2+^, Mn^2+^ and Ca^2+^, through dedicated transporters and sensors, especially when these ions are abundant in the parasite microenvironment (212).

### 5.2 Species-specific differences in metabolic regulation

*Leishmania* species exhibit distinct metabolic programs, especially in amino acid metabolism, which critically influence host modulation and nutrient competition. A global mass spectrometry–based metabolomic study of *L. major*, *L. mexicana*, and *L. donovani* cultured under standardized *in vitro* conditions, revealed significant species-specific metabolic differences independent of environmental factors (216, 217). Notably, *L. major* depleted nearly all available tryptophan, whereas *L. donovani* and *L. mexicana* consumed half and two-thirds, respectively (217). An enhanced parasite-mediated tryptophan depletion may impair host immunity, as reduced tryptophan availability suppresses T cell proliferation and function (218). In addition, *L. mexicana* exhibited high arginine consumption, while *L. major* and *L. donovani* preferentially catabolized arginine to arginic acid (217). Increased arginine flux during infection may favor polyamine synthesis, thus limiting nitric oxide-dependent killing. Together, these intrinsic, species-specific metabolic programs shape host immune response and disease outcomes during *Leishmania* infection.

## 6 Conclusion

Current treatment options for leishmaniasis, including antimonials, amphotericin B (Ambisome), and others, face significant challenges. While these agents effectively inhibit parasite replication, their clinical utility is hampered by host toxicity and emergence of drug-resistant strains (219). A promising alternative is to develop therapeutics that selectively target parasite-specific metabolic pathways and enzymes. Notably, vaccination of mice with centrin-deficient *L. mexicana* promotes enrichment of the pentose phosphate pathway, supporting NO production and classical macrophage activation (220). Targeting multiple metabolic pathways simultaneously may further enhance therapeutic efficacy, underscoring the need for a comprehensive understanding of *Leishmania*-driven host immunometabolic reprogramming.

Currently, there is no approved vaccine for human leishmaniasis. However, the observation that individuals who recover from infection generally acquire long-term immunity suggests vaccination is feasible. The challenge lies in identifying *Leishmania* antigens capable of eliciting strong and durable protective immune responses in the host. Reverse immunology approaches have identified peptides from *Leishmania* DLD and PEPCK, which are enzymes central to parasite energy metabolism and are key players at modulating T cell responses critical for disease resolution. Live attenuated vaccines candidates lacking either PEPCK (205) or DLD (11, 201) represent promising strategies for inducing protective immunity. Targeting parasite metabolic enzymes may also guide drug design. For example, indolamine 2,3-dioxygenase (IDO1) released by *Leishmania*-infected DCs can suppress inflammation and promote infection. Therefore, IDO1 inhibitors could reverse this effect and enhance effector T cell responses (221). Likewise, NMR metabolomic study of *Leishmania* infected macrophages identified several metabolites, including glycerophosphocholine, phosphocholine, creatine phosphate, and creatine as potential biomarkers and novel drug targets against the disease (222). An effective therapeutic strategy for leishmaniasis should both impair parasite survival and prime host immune cells to metabolically mount competent effector functions upon pathogen encounter. Continued advances in metabolomic techniques will be instrumental in elucidating host cell-*Leishmania* metabolic interactions that could inform the design of next-generation drugs and vaccines against the disease.
